# Nucleic acids and endosomal pattern recognition: how to tell friend from foe?

**DOI:** 10.3389/fcimb.2013.00037

**Published:** 2013-07-30

**Authors:** Eva Brencicova, Sandra S. Diebold

**Affiliations:** Peter Gorer Department of Immunobiology, Guy's Hospital, King's College LondonLondon, UK

**Keywords:** endosomal toll-like receptors, nucleic acids, innate immune activation, autoimmunity, pattern recognition

## Abstract

The innate immune system has evolved endosomal and cytoplasmic receptors for the detection of viral nucleic acids as sensors for virus infection. Some of these pattern recognition receptors (PRR) detect features of viral nucleic acids that are not found in the host such as long stretches of double-stranded RNA (dsRNA) and uncapped single-stranded RNA (ssRNA) in case of Toll-like receptor (TLR) 3 and RIG-I, respectively. In contrast, TLR7/8 and TLR9 are unable to distinguish between viral and self-nucleic acids on the grounds of distinct molecular patterns. The ability of these endosomal TLR to act as PRR for viral nucleic acids seems to rely solely on the mode of access to the endolysosomal compartment in which recognition takes place. The current dogma states that self-nucleic acids do not enter the TLR-sensing compartment under normal physiological conditions. However, it is still poorly understood how dendritic cells (DC) evade activation by self-nucleic acids, in particular with regard to specific DC subsets, which are specialized in taking up material from dying cells for cross-presentation of cell-associated antigens. In this review we discuss the current understanding of how the immune system distinguishes between foreign and self-nucleic acids and point out some of the key aspects that still require further research and clarification.

Viruses are dependent on the host metabolism for replication. As a consequence of this, the carbohydrate and lipid structures associated with viruses are very similar to those of the host and, therefore, do not represent suitable pathogen-associated molecular patterns (PAMP) for innate immune recognition. Instead, the immune system has evolved pattern recognition receptors (PRR) that recognize viral nucleic acids. Viral replication leads to the accumulation of viral replication intermediates in the cytoplasm of infected cells, which trigger cytoplasmic PRR such as the RIG-I-like receptors and various DNA sensors such as DAI, DExD/H box family helicases, AIM2-like receptors and cGAS (Kato et al., 2011; Keating et al., 2011; Sun et al., 2013; Wu et al., 2013). These cytoplasmic nucleic acid-sensing PRR initiate the local anti-viral response in the infected tissue. Cells of the innate immune system are recruited to the site of infection and together with local sentinels such as Langerhans cells and dermal dendritic cells (DC) in the skin and interstitial DC in other organs sense the infectious organism by means of PRR such as Toll-like receptors (TLR), which sample exogenous material for the presence of PAMP. PRR-mediated activation of DC by PAMP is thought to be crucial for the initiation of an adaptive immune response against the invading pathogen (Joffre et al., 2009). The requirement for direct activation of DC via PRR ensures that only DC which had direct contact to the infectious organism and present pathogen-derived antigens have the capacity to induce the differentiation of T cells into effector cells. However, in particular situations, indirect activation of DC via inflammasome-induced IL-1β seems to be sufficient to promote the induction of an adaptive immune response as observed upon live influenza A or herpes simplex virus infection in mice (Pang et al., 2013). Similarly, in house dust mite allergen-induced asthma direct TLR-mediated activation is dispensable (Hammad et al., 2009) and it is currently unclear which mechanism allows the immune system to repeal the need for direct activation of DC.

Recognition by cytoplasmic PRR partially depends on features of viral nucleic acids that are absent from mammalian nucleic acids such as 5′ triphosphate groups on short blunt-ended double-stranded RNA (dsRNA) structures recognized by RIG-I and long dsRNA structures recognized by MDA5 (Goubau et al., 2013). However, cytosolic DNA sensors are thought to be unable to distinguish between foreign and self DNA since it was shown that recognition is sequence-independent and unaffected by nucleic acid modifications such as methylation (Ishii et al., 2006; Stetson and Medzhitov, 2006). Furthermore, while DAI (also called DLM-1/ZBP1) was initially described as a Z-DNA-binding protein recognizing the rare left-handed Z-form of DNA associated with some viral structures, it was later demonstrated that the right-handed B-form of DNA as seen for self DNA and most commonly found under physiological conditions has the capacity to trigger a cytoplasmic DNA sensor upstream of the stimulator of interferon (IFN) genes (STING) (Schwartz et al., 2001; Ishii et al., 2006; Takaoka et al., 2007). In line with the hypothesis that cytoplasmic sensors are not specific for foreign DNA structures, self DNA induces polyarthritis in mice deficient for DNase II and type I interferon (IFN-I) receptor in a STING-dependent manner (Ahn et al., 2012). Similarly, TLR-mediated recognition of nucleic acids relies on molecular patterns that are present in self-nucleic acids as discussed later in more detail (Hornung et al., 2006; Pichlmair et al., 2006; Takaoka et al., 2007). This shows that the immune system has evolved mechanisms to prevent stimulation by self-nucleic acids under normal conditions, but that nucleic acid-sensing PRR can trigger innate immune activation in pathological situations. Thus, aberrant PRR activation by self-nucleic acids poses the risk of driving autoimmunity induction. In this review, we will discuss the current understanding of how the immune system distinguishes between viral vs. self-nucleic acids on the level of endosomal TLR and which mechanisms are involved in preventing autoimmunity induction driven by TLR-mediated sensing of self-nucleic acids under physiological conditions.

## Endosomal toll-like receptors and their agonists

**Figure 1:**
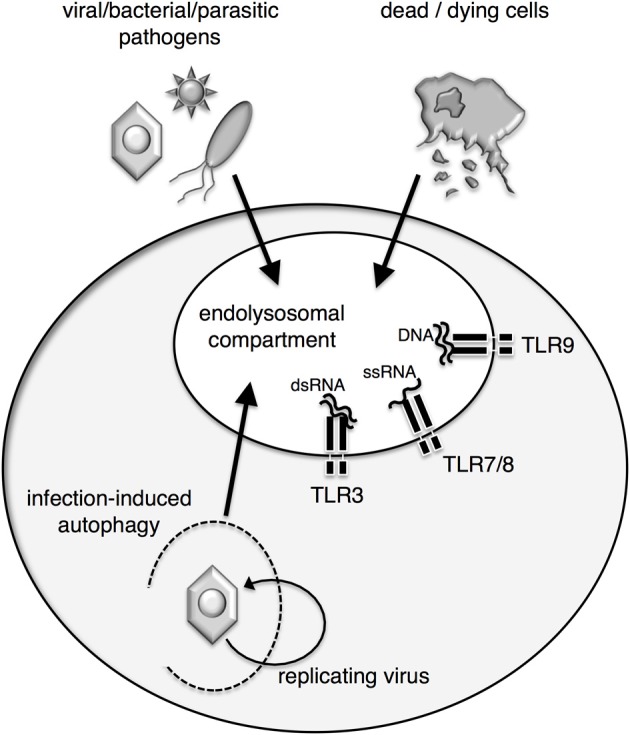
**Access of agonists to endosomal nucleic acid-sensing TLR**. Endosomal TLR are situated in the membrane of the endolysosomal compartment of APC and sample the content of these compartments for the presence of nucleic acid agonists. Pathogens or dead cells gain access to the compartment by endocytosis. Alternatively, infection-induced autophagy can shuttle viral nucleic acids and antigens into the endolysosomal compartment and allow for recognition of replicating virus within infected cells by endosomal TLR.

The family of endosomal nucleic acid-sensing TLR comprises TLR3, TLR7, TLR8, and TLR9 (Figure 1). While TLR7–9 form all endosomal TLR dependent on the endoplasmic reticulum (ER)-resident protein UNC93B1 for trafficking from the ER via the Golgi to the endolysosomal compartment (Figure 3A) (Du et al., 2000; Brinkmann et al., 2007; Kim et al., 2008). Other factors that affect the trafficking of endosomal TLR are the ER chaperone GP96 and PRAT4A (Randow and Seed, 2001; Takahashi et al., 2007; Yang et al., 2007b). For a comprehensive review on TLR trafficking see the review by Lee et al. (2012).

In addition, differential recruitment of adaptor protein complexes (AP) affects post-Golgi trafficking of endosomal TLR. While AP-2 recruitment by TLR9 enables its translocation from the plasma membrane to the endolysosomal compartment, TLR7 trafficking is independent of AP-2 and does not involve transient localization to the plasma membrane, but instead is dependent on AP-4 recruitment (Lee et al., 2013). TLR3 trafficking is again distinct from TLR7 and TLR9 and is independent of PRAT4A and not affected by mutations in UNC93B1 that impair TLR7 and TLR9 trafficking (Takahashi et al., 2007; Fukui et al., 2011). Interestingly, cell specific differences in endosomal TLR trafficking have also been reported. Trafficking of endosomal TLR in plasmacytoid DC (PDC) is dependent on the recruitment of AP-3 and LC-3, which direct the localization of TLR7 and TLR9 to an endosomal compartment specialized in inducing type I IFN in these natural IFN-producing cells (Sasai et al., 2010; Henault et al., 2012).

Once recruited to the endolysosomal compartment, the TLR are rendered functionally active upon cleavage by resident pH-dependent proteases (Ewald et al., 2008; Park et al., 2008). The requirement for cleavage upon arrival in the endolysosomal compartment is thought to prevent activation of the nucleic acid-sensing TLR by self-nucleic acids at other locations in the cell or at the cell surface and is regarded as one of the mechanism that prevent aberrant activation of these TLR by self-agonists under physiological conditions. However, once the TLR have been rendered functional through proteolytic cleavage, they cannot distinguish between viral and self-nucleic acids on the basis of structural differences with the exception of TLR3.

TLR3 recognizes viral dsRNA in a sequence-independent manner in form of genomic dsRNA or dsRNA replication intermediates present in virus-infected cells (Alexopoulou et al., 2001; Schulz et al., 2005). Genomic nucleic acid material from dsRNA viruses such as reoviruses and dsRNA replication intermediates as produced for example by alphaviruses consist of long stretches of dsRNA which represent molecular structures that are absent from uninfected eukaryotic cells. Only short structures of dsRNA are found among cellular RNA such as in the secondary clover-leaf structure of transfer RNA (tRNA) or in microRNA (miRNA). As such, viral dsRNA represents a bona fide PAMP that allows the immune system to distinguish between viral and self RNA molecules. A minimum length of 45 base pairs (bp) is thought to be required for binding to TLR3, but dsRNA molecules >90 bp show stronger induction of pro-inflammatory cytokines (Leonard et al., 2008; Liu et al., 2008; Jelinek et al., 2011). Surprisingly, 21 nucleotide-long small interfering RNA (siRNA) molecules have been reported to trigger TLR3-mediated activation *in vitro* and *in vivo* contradicting the requirement for dsRNA stretches longer than 45 bp (Kariko et al., 2004a,b; Kleinman et al., 2008). However, other reports failed to detect innate immune activation by siRNA molecules and, furthermore, miRNA and endogenously expressed hairpin siRNA (shRNA) are not immunogenic. These findings suggest that innate immune activation in response to siRNA may represent an experimental artifact possibly due to the formation of higher order structures by siRNA preparations. Similarly, messenger RNA (mRNA) was reported to induce TLR3 activation in experimental settings which may be a consequence of using highly pure mRNA preparations devoid of RNA-binding proteins allowing for the formation of higher order structures (Kariko et al., 2004c). Interestingly, functional TLR3 has been detected at the cell surface rather than in endosomes in human fibroblasts (Matsumoto et al., 2003). While the role of TLR3 at the cell surface of fibroblasts is entirely enigmatic, cell surface expression of this particular dsRNA-sensing PRR may not pose an increased risk with regard to autoimmunity induction, since dsRNA represents a bona fide PAMP absent from mammalian cells.

In contrast to TLR3, the other endosomal TLR namely TLR7, TLR8, and TLR9 are unable to distinguish between pathogen and self-nucleic acids on the basis of distinct molecular structures (Barbalat et al., 2011). Mouse TLR7 and human TLR7 and TLR8 serve as PRR for single-stranded RNA (ssRNA), whereas the functionality of mouse TLR8 is still somewhat obscure (Alexopoulou et al., 2012). In addition to viral ssRNA and synthetic uridine- or uridine/guanosine-rich oligoribonucleotides (ORN), there is a number of small immune modifiers developed by pharmaceutical companies with TLR7 and/or TLR8-stimulating activity such as the imidazoquinolines R848 and R837 (Smits et al., 2008). R837 is approved for topical application and is used for treatment of genital warts and basal cell carcinoma because of its TLR-mediated anti-viral and anti-tumouricidal activities, respectively (Wagstaff and Perry, 2007). When RNA was first discovered as the natural agonist of TLR7 and human TLR8, it was immediately obvious that the preference for guanosine/uridine (GU)-rich and uridine (U)-rich sequences by human and mouse ssRNA-sensing TLR, respectively, did not constitute a suitable basis for discrimination between pathogen-associated and self-nucleic acids (Diebold et al., 2004; Heil et al., 2004). Furthermore, mouse TLR7 was demonstrated to respond to ORN corresponding to eukaryotic mRNA sequences and purified mouse mRNA, showing that self-nucleic acid is immunostimulatory when delivered efficiently into the TLR7-sensing endosomal compartment (Diebold et al., 2004, 2006). Interestingly, mammalian RNA contains a high frequency of modifications such as methylated nucleosides or pseudouridines, which ablate TLR7 and TLR8 activation (Kariko et al., 2005). Such modifications are particularly abundant in mammalian tRNA and ribosomal RNA (rRNA) but less frequent in mammalian mRNA (Soll, 1971; Maden and Hughes, 1997). This could explain why total mammalian RNA, which contains a high percentage of RNA species with TLR-inhibitory modifications is not immunostimulatory whereas purified mammalian mRNA when delivered to the endosome in form of complexes with polycations such as polyethylenimine triggers TLR7-dependent innate immune activation (Koski et al., 2004; Kariko et al., 2005; Diebold et al., 2006). Further evidence that TLR7 and human TLR8 cannot discriminate between self and pathogen RNA on the basis of structural differences stems from findings that implicate a central role for the recognition of self RNA in the immunopathogenesis of autoimmune diseases such as systemic lupus erythematosus (SLE), psoriasis, rheumatoid arthritis, Sjörgen's syndrome and others (Theofilopoulos et al., 2011). Similarly, genetic modifications that lead to a duplication of the TLR7 gene or over-expression of transgenic TLR7 are associated with exacerbated lupus-like symptoms in murine models (Pisitkun et al., 2006; Deane et al., 2007). It is worth noting that TLR7 is located on the X chromosome and that females induce higher levels of IFN-I in response to TLR7 agonists (Berghofer et al., 2006), which could represent a major factor responsible for the higher prevalence of SLE in women.

A similar link to autoimmunity induction has been identified for TLR9 (Theofilopoulos et al., 2011). TLR9 has evolved as innate immune sensor for viral, bacterial, fungal and protozoan DNA and is also activated by synthetic oligodesoxyribonucleotides (ODN) with a phosphorothioate backbone and an unmethylated CpG motif (Bauer et al., 2001; Lund et al., 2003; Parroche et al., 2007; Nakamura et al., 2008). Since the immunostimulatory activity of ODN is dependent on the presence of an unmethylated CpG motif and since such motifs are more abundant in bacterial DNA than in mammalian DNA, it was thought for a long time that TLR9 is able to discriminate between pathogen and self DNA (Hemmi et al., 2000). However, further studies have questioned the requirement for a CpG motif for TLR9-mediated activation (Yasuda et al., 2006; Haas et al., 2008; Wagner, 2008). Using synthetic TLR9 agonists, it was discovered that phosphorothioate ODN have a much higher affinity to TLR9 than ODN with a natural phosphodiester backbone and that the latter exert TLR9 stimulatory activity independent of the presence of CpG motives (Haas et al., 2008). In direct contrast to this, when studying the activation of autoreactive B cells it became apparent that TLR9 stimulation is dependent not only on the presence of CpG motifs in mammalian DNA, but also on the hypomethylated status of the self-nucleic acids (Viglianti et al., 2003). While it is currently unclear how these conflicting results regarding the requirement for unmethylated CpG motives can be reconciled, it is evident that self DNA has the ability to induce TLR9 activation if it enters the endosomal compartment in which recognition takes place.

## How does the immune system discriminate between pathogen and self nucleic acids?

Once it became obvious that the nucleic acid structures recognized by TLR7, TLR8, and TLR9 do not constitute bona fide PAMP and that self-nucleic acids can trigger innate immune activation via these PRR under certain conditions, it was hypothesized that the endosomal localization of these TLR prevents activation by self-agonists under physiological conditions by refusing self-nucleic acids access to the compartment (Diebold et al., 2004; Heil et al., 2004; Barton et al., 2006; Barton and Kagan, 2009). According to this hypothesis, mechanisms that regulate the uptake of material containing pathogen vs. self-nucleic acids must be in place to prohibit innate immune activation in response to the latter.

In this context it is worth remembering that experimental systems often fail to mimic natural processes in all details with regard to innate immune activation by PAMP. In experimental systems, agonists for endosomal TLR such as CpG ODN, R848 and polyI:C for TLR9, TLR7/8, and TLR3 activation, respectively, are administrated in solution whereas ORN for stimulation of TLR7/8 are applied in form of complexes with polycations or cationic lipids such as polyethylenimine or DOTAP. In contrast, the natural agonists for endosomal TLR are an integral part of pathogens including viruses or virus-infected host cells and as such are not directly accessible for receptor binding neither at the cell surface nor in the endolysosomal compartment. Consequently, pathogen- and host cell-derived material associated with pathogen nucleic acids have to be taken up into the endolysosomal compartment, where degradation allows the nucleic acids to become available for TLR binding. Thus, synthetic mimics of viral nucleic acids added directly to cells *in vitro* or used as adjuvants *in vivo* in mouse models are unsuitable to investigate mechanisms that allow the immune system to distinguish pathogen from self-nucleic acids.

Nevertheless, “naked” pathogen-derived and self-nucleic acids may be present in the extracellular space upon release from damaged microbes or disintegrating infected and uninfected host cells. Under physiological conditions, however, natural “naked” nucleic acids are degraded by extracellular DNases and RNases before they can access the endolysosomal compartment. Where such degradation is defective, the danger emanating from released self DNA becomes evident as seen in SLE patients carrying mutations in DNase I and in DNase I-deficient mice, which develop a lupus-like disease (Napirei et al., 2000; Yasutomo et al., 2001). Interestingly, receptors for the uptake of nucleic acids such as scavenger receptors and C-type lectin receptors (CLR) have been described and seem to play a role in the uptake of synthetic nucleic acid TLR agonists such as CpG ODN and polyI:C (Zhu et al., 2001; Limmon et al., 2008; Lahoud et al., 2012). However, synthetic nucleic acid TLR agonists with a phosphorothioate backbone and dsRNA homopolymers as commonly used for experimental TLR activation are more resistant to enzymatic degradation than natural nucleic acids and a relevant role of nucleic acid-binding scavenger receptors in delivering natural nucleic acids into the TLR-sensing endolysosomal compartment under non-pathological conditions is highly questionable. Thus, in order to understand how pathogen-associated nucleic acids gain access to the TLR-sensing endolysosomal compartment while self-nucleic acids are excluded from entering, it is necessary to take into account which pathways are involved in uptake of pathogens and dead cell material.

## How is the uptake of pathogens and dead cell material mediated?

**Figure 2:**
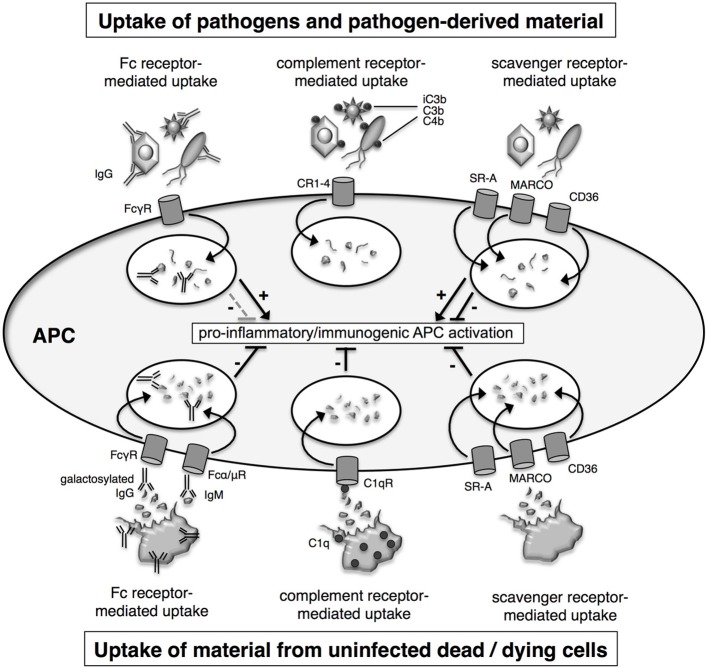
**Mechanisms for uptake of pathogens and dead cells**. Material from pathogens and dead cells is taken up via the same uptake mechanisms, which include Fc receptor-, complement receptor- and scavenger receptor-mediated uptake. Fc receptor- and complement receptor-mediated uptake require the prior opsonisation of the cargo with antibodies and complement factors, respectively. APC activation is tightly regulated and influenced both by the source of the material and uptake route. Uptake of material from dying uninfected cells induces a number of regulatory pathways which attenuate pro-inflammatory immunogenic antigen-presenting cells (APC) activation. In contrast, uptake of pathogens or pathogen-derived material as associated with infected dying cells leads to pro-inflammatory immunogenic activation of APC.

Since nucleic acids are typically not available at the surface of pathogens, they are unlikely to play a role in pathogen uptake. Furthermore, it has been shown that uptake of TLR agonists is independent of TLR9, even in soluble form such as CpG ODN. During infections, cells of the innate immune system internalize pathogens for sampling by endosomal TLR by a number of different ways: (1) by receptor-mediated endocytosis in response to binding to scavenger receptors, (2) by phagocytosis which is promoted by the complement system through opsonisation, and (3) by Fc receptor (FcR)-mediated uptake upon opsonisation with antibodies (Figure 2). Similarly, material from infected and uninfected dead cells is taken up by specialized cell types such as macrophages and DC involving scavenger receptor-, complement- and antibody-mediated uptake (Figure 2).

### Scavenger receptor-mediated uptake

Uptake of pathogens and pathogen-derived material is mediated by scavenger receptors that are expressed among other cell types by phagocytic macrophages and antigen-presenting DC. Scavenger receptors encompass a group of structurally unrelated transmembrane surface molecules with relatively promiscuous ligand binding characteristics such as scavenger receptor A (SR-A), MARCO and CD36. This promiscuity allows scavenger receptors to mediate uptake of a wide range of pathogens including gram-positive and gram-negative bacteria, yeast, viruses and parasites (Mukhopadhyay and Gordon, 2004). However, due to the observed redundancy and the promiscuous and overlapping ligand-binding characteristics of scavenger receptors, little is known about their natural ligands and the structural basis for ligand binding. Interestingly, scavenger receptors are also involved in the uptake of dead cell material by both macrophages and DC (Peiser et al., 2002; Harshyne et al., 2003; Parlato et al., 2010). In case of macrophages, clearance of dead cells is involved in re-establishment of tissue homeostasis whereas DC ingest cellular material to instruct and regulate T cell responses against cellular and cell-associated antigens. Due to these functional differences, the uptake of pathogens and dead cell material differs quantitatively between macrophages and DC. In addition, qualitative differences in the pathways that mediate uptake by these antigen-presenting cell types also exist. For example, it has been shown that, unlike macrophages, DC acquire cell-associated antigen from live cells via a SR-A-dependent uptake mechanisms (Harshyne et al., 2003). However, the composition of the cellular material ingested by DC through “nibbling” on live cells is unknown and it would be interesting to determine whether cellular nucleic acids are present in the cellular material that is taken up or whether specific species of cellular nucleic acids are excluded.

In macrophages, the scavenger receptors MARCO and SR-A are involved in uptake of CpG ODN and influence TLR9-mediated IL-12 induction with MARCO enhancing and SR-A reducing its production (Jozefowski et al., 2006). Interestingly, the binding to SR-A is dependent on the presence of serum, suggesting that CpG ODN may have to be available in form of complexes with nucleic acid-binding proteins in order to bind SR-A (Jozefowski et al., 2006). Similarly, the serum factor granulin or its precursor pro-granulin binds to CpG ODN and mediates uptake into the endolysosomal compartment probably by a sortilin-dependent pathway (Park et al., 2011). Macrophages from MARCO and SR-A deficient mice show the same opposing alterations in IL-12 induction in response to the TLR4 agonist lipopolysaccharide suggesting that the modulation of TLR activation by scavenger receptors is not restricted to endosomal nucleic acid-sensing TLR (Jozefowski et al., 2005).

### Complement-mediated phagocytosis

In addition to scavenger receptor-mediated uptake, pathogens and dead cell material can be ingested via complement receptor-mediated uptake upon opsonisation with complement factors. In contrast to live cells, which have the ability to actively remove deposits of the complement cascade on their cell surface, dead cells and pathogens are unable to do so and become opsonised by complement factors (Trouw et al., 2008). For example, the complement component C1q binds to antibodies complexed with antigen. This includes IgG and IgM antibodies bound to soluble antigens, pathogens and dead cells. Interestingly, C1q has also been shown to bind DNA and RNA, which is exposed on the surface of dying cells in late apoptosis and is involved in the degradation of DNA in cooperation with DNase I (Gaipl et al., 2004; Palaniyar et al., 2004). Thus, binding and removal of cellular DNA from dead cells by C1q presents one mechanism that prevents the activation of the innate immune system by self-nucleic acids. Furthermore, C1q inhibits NFκ B activation in response to TLR stimulation, reducing the induction of pro-inflammatory cytokines by monocytes, macrophages and DC (Fraser et al., 2009). The important regulatory role of C1q in preventing innate immune activation by dead cells accumulating in the tissue is underpinned by the finding that C1q deficiency leads to autoimmunity induction in humans and mice resulting in SLE and SLE-like symptoms, respectively (Schur, 1977; Botto et al., 1998). Defects in other proteins that are involved in the removal of cell remains such as mutations in serum amyloid P, milk fat globule-epidermal growth factor 8 and TAM receptor protein tyrosinase kinase family also lead to lupus-like syndromes (Bickerstaff et al., 1999; Paul and Carroll, 1999; Lu and Lemke, 2001; Hanayama et al., 2004). Interestingly, not all defects in complement proteins participating in the removal of dead cells from the tissue lead automatically to SLE-like symptoms, since deficiencies in other complement components such as Mannan-binding lectin (MBL) and C3 affect uptake of dead cells but do not promote autoimmunity induction (Takizawa et al., 1996; Sekine et al., 2001; Stuart et al., 2005). Taken together, these findings support the hypothesis that the ability of C1q to instigate DNA degradation and to regulate TLR-mediated NFκ B activation may represent characteristics of C1q function that are crucial for the prevention of endosomal TLR activation by self-nucleic acids and autoimmunity induction.

### FcR-mediated uptake

Similar to opsonisation with complement, pathogens can be opsonised with antibodies mediating uptake via FcR on macrophages and DC. As for receptor- and complement-mediated uptake, antibody-mediated uptake is not restricted to pathogens and is also observed for dead cells. In particular, IgM was shown to bind to oxidation-specific epitopes exposed on late apoptotic cells (Chang et al., 1999). Interestingly, IgM-mediated uptake of apoptotic cells via Fcα/μ R down-modulates inflammatory responses by regulating APC activation and inhibits inflammatory and autoimmune diseases such as arthritis and SLE, respectively (Kim et al., 2003; Chen et al., 2009b; Jiang et al., 2011; Notley et al., 2011). Opsonisation with IgG and uptake by Fcγ receptor (Fcγ R) can either promote or down-modulate activation of antigen-presenting cells depending on which Fcγ R family members mediates uptake. Independent of activation, antigen presentation is much more efficient for antigens taken up in form of immune complexes via Fcγ R than for antigens ingested by pinocytosis (Ivan and Colovai, 2006).

In addition to promoting uptake of pathogens and dead cells through opsonisation, antibodies bound to antigen also trigger the complement cascade and thus promote the killing of pathogens and advance the phagocytosis of pathogens and dead cells through further opsonisation with complement factors creating a potent feedback loop.

### Differences in ingesting pathogens vs. dead cell material

None of the discussed uptake mechanisms for pathogens differ fundamentally from those mediating uptake of dead cells. While C1q- and IgM-mediated uptake of apoptotic cells is associated with induction of regulatory pathways and crucial for preventing autoimmunity induction, their binding to dead cells is not exclusive and they play an equally important role in uptake of pathogens. This seems entirely reasonable since both dead cells and pathogens represent entities that have to be removed in order to restore tissue homeostasis. However, the hazards emanating from uninfected dead cells are very different from those associated with pathogens and infected dying cells and the immune system has to be able to distinguish the two scenarios in order to prevent autoimmunity induction. The discrimination between pathogen and cellular nucleic acids is most crucial for APC with the capacity to prime or promote antigen-specific T cell responses in response to activation via nucleic acid sensing TLR.

## DC and the discrimination between pathogen and self nucleic acids

PDC and specific DC subsets specialized in cross-priming of viral antigen-specific CD8 T cells seem to play particular but distinct roles during virus infections (Belz et al., 2009). PDC are a cell type specialized in virus recognition via TLR7 and TLR9 (Bao and Liu, 2013). They produce high systemic levels of IFN-I upon recognition of viral ssRNA or viral DNA and are, therefore, also known as natural IFN-producing cells (Bao and Liu, 2013). High levels of systemic IFN-I are a hallmark of viral infection and influence the anti-viral immune response on many levels (Levy et al., 2011). IFN-I drives the differentiation of monocytes into inflammatory DC, promotes the T cell stimulatory activity of APC, supports T helper cell-independent class switching of B cells, skews the T helper response toward a Th1 phenotype and aids the induction of cytotoxic T lymphocyte (CTL) responses by directly acting on CD8 T cells (Le Bon and Tough, 2008). However, IFN-I production in response to virus infection is not necessarily dependent on PDC (Swiecki and Colonna, 2010). While it is accepted that PDC are specialized in virus recognition and IFN-I production, the role of PDC as antigen-presenting cells during virus infection is less well understood. There are conflicting results on their endocytic activity and their ability to present exogenous antigens and it is evident that PDC differ from other DC subsets with regard to the uptake and processing of exogenous antigens (Villadangos and Young, 2008). However, PDC clearly take up virus particles. Viruses such as influenza virus enter PDC via receptor-mediated uptake into the TLR-sensing endolysosomal compartment, which is their natural infection route (Patterson et al., 1979; Londrigan et al., 2012). In contrast, fusogenic viruses such as Coxsackie virus and foot-and-mouse-disease virus are dependent on the presence of anti-viral antibodies and complex formation for uptake into endosomes via Fcγ R expressed by PDC (Guzylack-Piriou et al., 2006; Wang et al., 2007). In addition to the recognition of genomic viral material, PDC can become activated by viral ssRNA replication intermediates, which accumulate in the cytoplasm upon infection and are then shuttled into the endolysosomal compartment for recognition by TLR7 by autophagy (Lee et al., 2007). The initiation of virus-induced autophagy is driven by cytoplasmic PRR and hence cytoplasmic self-nucleic acids are not subject of translocation to the endolysosomal compartment in uninfected cells (Tal and Iwasaki, 2009). Thus, under physiological conditions, PDC usually do not encounter cellular nucleic acids making a distinction between self-versus pathogen nucleic acid dispensable for this cell type. However, once the regulatory mechanism that prevent the release and accumulation of nucleic acids from dead cells fail and allow for complex formation of cellular RNA and DNA with antibodies or the antimicrobial peptide LL37, PDC become a driving force for autoimmune induction as observed in SLE and psoriasis, respectively (Lovgren et al., 2004; Barrat et al., 2005; Lande et al., 2007; Ganguly et al., 2009).

**Figure 3:**
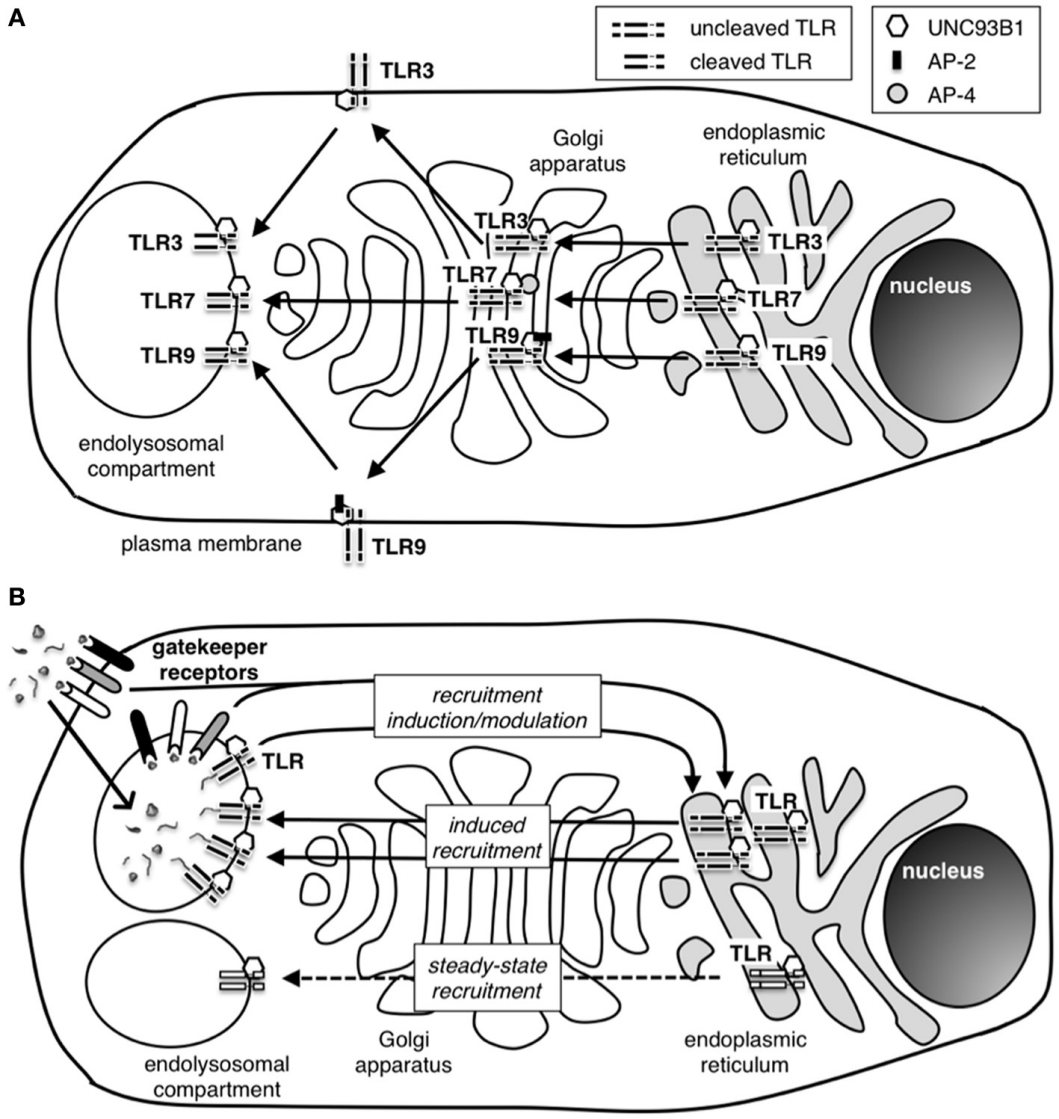
**How are nucleic acid-sensing TLR recruited to the endolysosomal compartment? (A)** Nucleic acid sensing TLR traffic to the endolysosomal compartment from the endoplasmic reticulum (ER) via the Golgi apparatus. The chaperone protein UNC93B1 is necessary for successful migration of all three endosomal TLR, and adaptor protein (AP) complexes AP-2 an AP-4 are additionally required during the shuttle process of TLR9 and TLR7, respectively. TLR9, but not TLR7, is transiently located at the cell plasma membrane before reaching the endolysosome. Similarly, TLR3 has been detected at the cell surface in specific cell types. The reason for these differences in trafficking pathways for endosomal TLR is currently unknown. While in the ER and passing through the Golgi, TLR remain in an uncleaved state and only undergo cleavage to attain functional activation after arrival in the endolysosome. The requirement for cleavage is thought to be a protective mechanism to avoid unwanted TLR activation outside the endolysosomal compartment. **(B)** The induction and regulation of TLR recruitment to the endolysosomal compartment is still poorly understood. A small number of TLR have been detected in the endolysosomal compartment in the absence of activating stimuli suggesting the presence of a self-perpetuating low-frequency shuttling process of TLR to the endolysosome (steady-state recruitment). During infection, high frequency recruitment of TLR to the endolysosome takes place (induced recruitment). Induced recruitment is likely to be initiated by PAMP-activated TLR and/or other PRR. However, gatekeeper receptors controlling pathways such as the CD24-Siglec or CLR-Syk pathway have the ability to modulate and maybe even induce TLR recruitment to the endolysosomal compartment. The presence of gatekeeper receptors with the ability to distinguish specific signals from pathogens or uninfected host cells and to promote or regulate TLR recruitment is likely to play an important role in protecting the host from innate autoimmune activation.

Specific DC subsets such as mouse CD8α^+^ DC, the related CD103^+^ DC and human BDCA3^+^ DC are specialized in the uptake of cellular material from dying cells and cross-present cell-associated antigen to CD8 T cells. While immature CD8α-like DC are involved in peripheral tolerance induction, activated CD8α-like DC prime CTL responses against cell-associated antigens and thereby play a crucial role in anti-viral immunity (Joffre et al., 2012). The cellular material that is taken up by this specialized DC subset is sampled in the endolysosomal compartment in which TLR activation takes place. CD8α-like DC express TLR3 and TLR8 and, therefore, can detect viral dsRNA and theoretically also ssRNA associated with virus-infected cells (Schulz et al., 2005). It is still entirely unclear how CD8α-like DC avoid the activation by cellular RNA. The presence of gatekeeper receptors with the ability to control or modulate the recruitment of nucleic acid-sensing TLR to the endolysosomal compartment of CD8α-like DC could play a role in avoiding the activation of these cells in response to self-nucleic acids from uninfected dying cells. The recruitment of nucleic acid-sensing TLR to the endolysosomal compartment could be initiated by gatekeeper receptors upon sensing of non-nucleic acid PAMP or damage-associated molecular patterns (DAMP) present in infected cells but absent from uninfected cells (Figure 3B). Alternatively, CD8α-like DC may be very selective regarding the cellular material that they ingest in order to reduce the uptake of self-nucleic acids. Mouse CD8α^+^ DC and human monocyte-derived DC have been shown to “nibble” on apoptotic cells rather than ingesting whole cells (Schulz and Reis E Sousa, 2002; Harshyne et al., 2003). It is currently unclear what cellular components are present in the cellular fragments that are taken up by CD8α-like DC. By restricting the endocytosed material to membrane fragments, it may be possible for CD8α-like DC to favor uptake of viral components that associate with the plasma membrane as part of virus assembly and/or viral budding processes and to avoid uptake of cellular nucleic acids. A better understanding of the mechanisms underlying the uptake of cellular material by CD8α-like DC could provide vital insight into how discrimination between viral and self-nucleic acids is achieved by these specialized APC.

## What mechanisms aid in the discrimination between viral and cellular nucleic acids?

Since the uptake of pathogens and dead cells is not fundamentally different, it is very likely that additional mechanisms have to be in place to favor the recognition of viral nucleic acids and prevent the recognition of self-nucleic acids. Mechanisms that would allow the innate immune system to discriminate between self and viral nucleic acids could be based on the following characteristics: (1) as discussed above total cellular nucleic acids contain enough modified RNA species such as tRNA and rRNA which inhibit the activation of endosomal TLR in the presence of stimulatory self-nucleic acid such as mRNA; (2) sequestration of cellular nucleic acids through binding to cellular components may prevent binding to endosomal TLR and/or (3) the recruitment of nucleic acid-sensing TLR to the endolysosomal compartment or their functional activation by cleavage may be regulated by gatekeeper molecules sensing PAMP and/or DAMP absent from uninfected dead cells or DAMP absent from infected dead cells. The evidence for and the implications of the existence of these mechanisms and their involvement in the discrimination between pathogen and self-nucleic acids are discussed below.

**(1)** Modified cellular nucleic acids clearly can inhibit endosomal TLR activation by self-nucleic acids as discussed earlier (Kariko et al., 2005). However, it is less clear how TLR activation by viral nucleic acids upon uptake of material from infected cells is possible in the presence of modified cellular nucleic acids. It is unclear whether or not viral nucleic acid species associated with material from infected cells directly compete with cellular nucleic acids including modified nucleic acid species for binding to endosomal TLR. If this is the case, virus-derived nucleic acids have to be present in relative excess to trigger a response. The ratio between viral and cellular nucleic acids present inside infected cells is highly variable between viruses. While little is known about quantitative and qualitative differences in binding of viral vs. cellular nucleic acids to TLR, relative binding of RNA to polyribosomes in infected cells has been quantified. Quantitative analysis of viral vs. cellular RNA for Semiliki forest virus, herpes simplex virus and moloney murine leukaemia virus excluded direct competition as a means for preferential translation of viral RNA in infected cells (Tuomi et al., 1975; Stringer et al., 1977; Guttman-Bass et al., 1980). Thus, it is unlikely that direct competition between viral and cellular RNA in infected cells forms the general basis for preferential recognition of viral RNA associated with infected cells by endosomal TLR.

**Figure 4:**
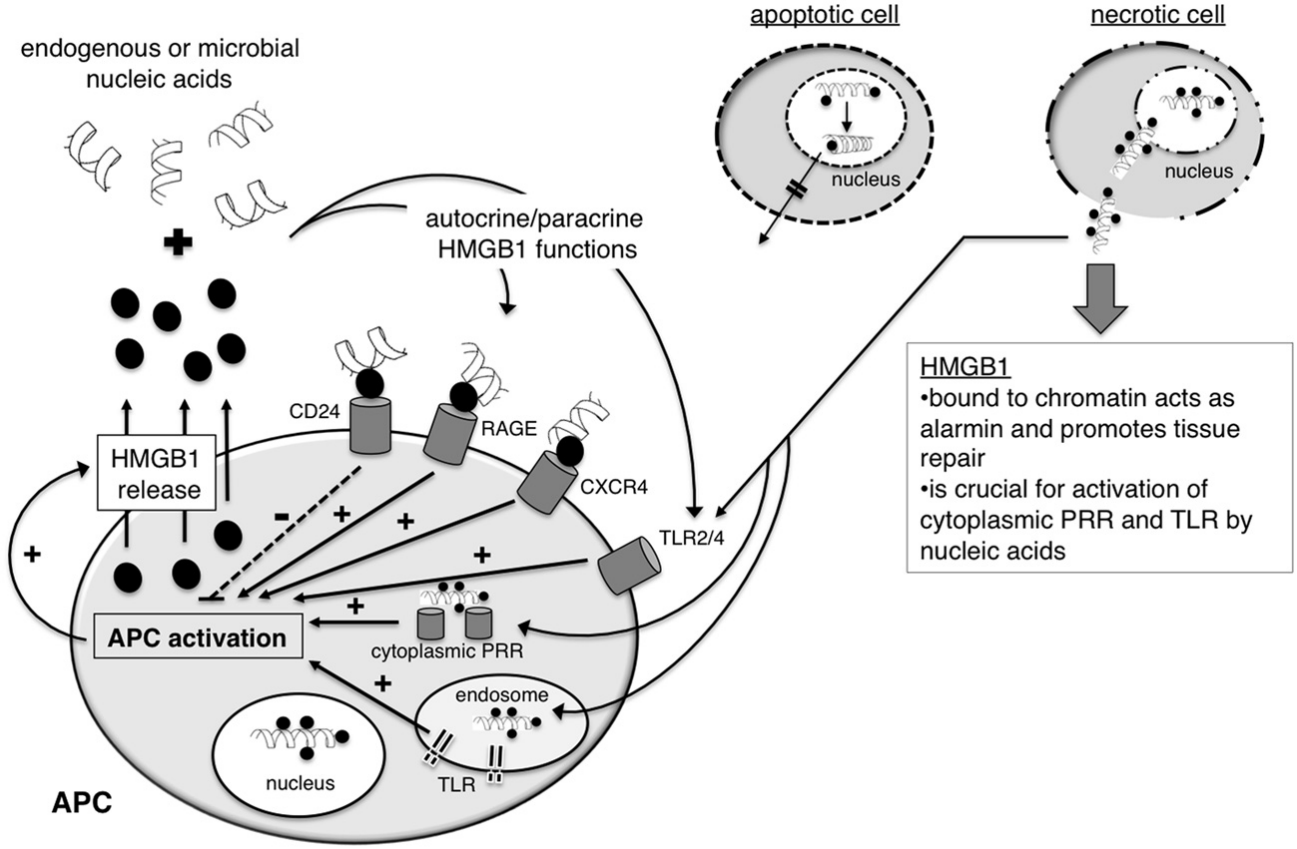
**The nuclear protein HMGB1 holds diverse functions in health and disease**. HMGB1 is a ubiquitous nuclear DNA-binding protein. During apoptosis, the binding of HMGB1 to chromatin tightens, preventing the release of the cells' nucleic acids into surrounding tissue. In contrast, upon primary necrosis, complexes of HMGB1 and chromatin are released into the extracellular space. Such complexes act as alarmins, supporting tissue repair, and they have the capacity to stimulate cytoplasmic nucleic-sensing PRR and TLR. It is currently not entirely understood what factors favor the activation of a regulatory rather than an immunogenic response by HMGB1. HMGB1 is also released from antigen-presenting cells (APC) upon activation via PRR. After binding to nucleic acids in surrounding tissue, HMGB1 can act in an autocrine and paracrine fashion, signaling via a range of receptors including TLR2/4, RAGE, CXCR4, and CD24.

**(2)** One example for sequestration of cellular DNA in dying cells is high-mobility group protein B1 (HMGB1). HMGB1 binds to DNA and interacts with nucleosomes, transcription factors and histones. During early apoptosis HMGB1 binds tightly to chromatin within the dying cell as a consequence of underacetylation and, thereby, prevents the release of HMGB1 and cellular DNA even after the cells undergo secondary necrosis (Scaffidi et al., 2002) (Figure 4). Thus, HMGB1 represents a prime example for a molecule sequestering cellular nucleic acids in order to prevent unwanted inflammation and immune responses in response to cell death in the absence of infection.

However, HMGB1 also plays multiple roles in influencing the induction of immune responses mediated through a number of different receptors it can bind to. Upon release, HMGB1 can signal through TLR2, TLR4, the receptor for advanced glycation endproducts (RAGE), CD24, Thrombin and CXCR4 (Yanai et al., 2012). HMGB1 is released by monocytes, macrophages and DC in response to TLR stimulation (Dumitriu et al., 2005). Autocrine/paracrine HMGB1 plays a crucial role in DC mobilization. Binding of HMGB1 to RAGE on DC controls the homing of activated DC to the draining lymph node (Manfredi et al., 2008). Interestingly, engagement of RAGE by HMGB1 also promotes the induction of IFN-I by PDC in response to CpG ODN (Tian et al., 2007). However, as observed for TLR9, the binding of ODN to HMGB1 seems to be affected by the sugar backbone with natural phosphodiester ODN showing little binding in contrast to artificial phopshorothioate ODN. Furthermore, HMGB proteins have been described as universal sentinels for nucleic acids, since HMGB1-3 bind different nucleic acid species and promote the activation of cytoplasmic and endosomal nucleic acid-sensing PRR (Yanai et al., 2009). The activation of cytoplasmic and endosomal nucleic acid-sensing PRR is severely impaired in the absence of HMGB proteins and the authors, therefore, postulated that binding of RNA and DNA to HMGB proteins may be a general prerequisite for activation of nucleic acid-sensing PRR. Alternatively, nucleic acid-binding to HMGB proteins may support ligand recognition by preventing nucleic acid degradation. In this context, it is interesting to note that DC release HMGB1 upon activation and thereby may aid the recognition of viral nucleic acids in an inflammatory context (Dumitriu et al., 2005).

The seemingly opposing roles of HMGB1 in protecting innate immune activation in response to cellular DNA and promoting DC activation upon secretion in response to TLR stimulation are difficult to reconcile. Furthermore, since neither HMGB1 nor the ssRNA- and DNA-sensing TLR are able to discriminate between pathogen-derived and self-nucleic acids, the promotion of endosomal TLR activation by nucleic acids bound to HMGB proteins has the potential for disastrous consequences with regard to autoimmunity induction. In support of this notion are the findings that elevated expression levels of HMGB1 have been detected in serum and in kidney of SLE patients, suggesting that HMGB1 plays a role in autoimmune pathology (Jiang and Pisetsky, 2008; Qing et al., 2008). Thus, the release of stimulatory nucleic acid-HMGB1 complexes from necrotic cells may have to be regarded as a pathological situation. However, a physiological role for such complexes cannot be entirely excluded and it was argued that these complexes represent an alarmin with the capacity to promote repair in damaged tissue (Yang et al., 2007a). Interestingly, HMGB1 also binds to CD24, which triggers the regulatory Siglec G pathway and, thereby, suppresses immune activation in response to HMGB1-TLR4 signaling (Chen et al., 2009a). While the CD24-Siglec pathway protects the host from responding to pathological cell death, responses to PAMP are unaffected by this pathway. As such, the CD24-Siglec pathway may represent one example for a pathway with gatekeeper function that allows the immune system to discriminate between endogenous alarmins vs. pathogen-derived PAMP.

**(3)** Gatekeeper receptors with the ability to detect PAMP and/or DAMP absent from uninfected cells are involved in the recruitment or the cleavage of nucleic acid-sensing TLR to the endolysosomal compartment would represent an additional powerful mechanism for preventing the recognition of self-nucleic acids upon uptake of cellular material from uninfected cells (Figure 3B). While it is established that nucleic acid-sensing TLR have to be recruited to the endolysosomal compartment upon uptake of pathogens or cellular material, the exact mechanisms of the recruitment remain unexplored. Initially, it was thought that TLR9 is located in the ER in unstimulated cells and is recruited to the endolysosomal compartment only after uptake of TLR9 agonist (Latz et al., 2004). More recent findings indicate that low level of trafficking of nucleic acid-sensing TLR via the Golgi to the endolysosomal compartment takes place in the steady state and that these few TLR molecules may initiate TLR recruitment upon stimulation with nucleic acids (Ewald et al., 2008; Park et al., 2008; Chockalingam et al., 2009; Blasius and Beutler, 2010). This model is supported by the finding that transgenic TLR-GFP is cleaved and transported to the endolysosomal compartment in unstimulated cells (Avalos et al., 2013). Interestingly, TLR4 activation induces the recruitment of nucleic acid-sensing TLR to the endolysosomal compartment, showing that other pathways can influence TLR-mediated detection of nucleic acids (Kim et al., 2008). Additional evidence that gatekeeper receptors may regulate the recruitment and/or cleavage of endosomal TLR stems from findings demonstrating that CpG ODN-mediated TLR9-independent activation of the Src family pathway at the cell surface of antigen-presenting cells is crucial for the subsequent activation of NFκ B via TLR9 (Sanjuan et al., 2006). This study further indicated the indirect association of TLR9 and Syk downstream of CpG-mediated activation of the Src family pathway. Possible candidates for Syk recruitment include ITAM- and ITIM-containing CLR, which play a role in antigen-processing and -presentation in DC and other myeloid cells (Kerrigan and Brown, 2010). While there is no direct evidence for CLR acting as gatekeeper molecules with the capacity to control the recruitment or the cleavage of nucleic acid-sensing TLR, CLR are known to influence TLR-mediated immune activation of antigen-presenting cells (Dennehy et al., 2008; Ferwerda et al., 2008; Meyer-Wentrup et al., 2009; Xaplanteri et al., 2009; Jahn et al., 2010; Eberle and Dalpke, 2012).

CLR comprise a very heterogeneous family of molecules with a carbohydrate recognition domain, which mediates binding to carbohydrate structures of pathogen- or self-origin (Robinson et al., 2006). The CLR DC-SIGN has been shown to down-modulate activation in response to TLR stimulation via Raf-dependent acetylation of NFκ B (Gringhuis et al., 2007). DC-SIGN is a CLR that has evolved to mediate cellular interactions by binding to ICAM-2 and ICAM-3. However, a number of mannose-expressing pathogens, such as mycobacterium tuberculosis and HIV, bind to DC-SIGN and down-modulate TLR-mediated immune activation to their own advantage. While DC-SIGN clearly has the ability to influence TLR-mediated activation, it is primarily involved in the recognition of self-carbohydrate moieties as part of intercellular interactions. It blunts rather than promotes TLR-mediated activation and is, therefore, an unlikely candidate for initiating the recruitment of TLR to the endolysosomal compartment.

Another pathway with the ability to detect PAMP which has been shown to influence TLR-mediated immune activation is the complement system. The anaphylatoxins C3a and C5a increase NFκ B activation and lead to enhanced induction of pro-inflammatory cytokines such as IL-6 and TNFα in mouse and human DC (Peng et al., 2006, 2008; Lalli et al., 2007; Li et al., 2012). However, regulatory effects of anaphylatoxins have also been described resulting in reduced induction of IL-12, IL-23, and IL-27 (Hawlisch et al., 2005; Lalli et al., 2007). Recently, it was shown that N-glycan galactosylation of IgG1 antibody promotes concurrent signaling via the CLR dectin-1 and the inhibitory Fcγ RIIB receptor and, thereby, exerts anti-inflammatory influence on C5a-mediated inflammatory responses (Karsten et al., 2012). The frequency of galactosylated serum IgG decreases under pro-inflammatory conditions such as during infection or in autoimmune disease (Karsten et al., 2012). Thus, the regulation of N-glycan galactosylation of IgG seems to represent an additional mechanism to dampen unwanted pro-inflammatory responses in an uninfected host.

The crosstalk between complement and TLR activation is a perfect example for how two independent PRR systems that are triggered by separate sets of PAMP can converge and provide positive feedback for increased innate immune activation. However, it is still poorly understood why complement activation enhances cytokine induction in response to PRR activation in some situations and dampens the response in others. Further insight into the factors that determine under which conditions complement promotes or suppresses TLR-mediated immune activation may lead to a better understanding of how innate immune activation in response to sterile cell death and the release of potentially stimulatory self-nucleic acids is avoided.

## Conclusion

In conclusion, we put forward the hypothesis that the sensing of naked ssRNA and DNA, which is not associated with pathogen-derived material including non-nucleic acid PAMP, represents a precarious feature of the immune system. It doesn't allow for the discrimination between pathogen-associated vs. self-nucleic acids and, therefore, has the potential to lead to autoimmunity. Hence, any mechanisms that allow for or promote the recognition of naked ssRNA and DNA such as in form of immune complexes should be regarded as non-physiological events. Furthermore, we propose that the discrimination between pathogen-derived and self-nucleic acids can be based on at least three separate mechanisms alone or in combination: (1) avoidance of uptake of self-nucleic acids in a form that allows TLR binding, (2) association of self-nucleic acids with cellular components that prevent TLR binding and (3) the presence of gatekeeper receptors, which control the recruitment of TLR to the endolysosomal compartment. Identification of the mechanisms that favor the recognition of pathogen-associated nucleic acids and limit TLR-mediated activation in response to self-nucleic acids will be instrumental for developing more refined strategies to exploit activation of endosomal TLR in the context of vaccines and tumor immunotherapy and for gaining a better understanding of the processes that can lead to autoimmunity induction.

### Conflict of interest statement

The authors declare that the research was conducted in the absence of any commercial or financial relationships that could be construed as a potential conflict of interest.
